# Atherosusceptible Shear Stress Activates Endoplasmic Reticulum Stress to Promote Endothelial Inflammation

**DOI:** 10.1038/s41598-017-08417-9

**Published:** 2017-08-15

**Authors:** Keith A. Bailey, Fawaz G. Haj, Scott I. Simon, Anthony G. Passerini

**Affiliations:** 1 Department of Biomedical Engineering, University of California, Davis, CA USA; 2 Department of Nutrition, University of California, Davis, CA USA; 3 Department of Internal Medicine, University of California, Davis, CA USA

## Abstract

Atherosclerosis impacts arteries where disturbed blood flow renders the endothelium susceptible to inflammation. Cytokine activation of endothelial cells (EC) upregulates VCAM-1 receptors that target monocyte recruitment to atherosusceptible regions. Endoplasmic reticulum (ER) stress elicits EC dysregulation in metabolic syndrome. We hypothesized that ER plays a central role in mechanosensing of atherosusceptible shear stress (SS) by signaling enhanced inflammation. Aortic EC were stimulated with low-dose TNFα (0.3 ng/ml) in a microfluidic channel that produced a linear SS gradient over a 20mm field ranging from 0–16 dynes/cm^2^. High-resolution imaging of immunofluorescence along the monolayer provided a continuous spatial metric of EC orientation, markers of ER stress, VCAM-1 and ICAM-1 expression, and monocyte recruitment. VCAM-1 peaked at 2 dynes/cm^2^ and decreased to below static TNFα-stimulated levels at atheroprotective-SS of 12 dynes/cm^2^, whereas ICAM-1 rose to a maximum in parallel with SS. ER expansion and activation of the unfolded protein response also peaked at 2 dynes/cm^2^, where IRF-1-regulated VCAM-1 expression and monocyte recruitment also rose to a maximum. Silencing of PECAM-1 or key ER stress genes abrogated SS regulation of VCAM-1 transcription and monocyte recruitment. We report a novel role for ER stress in mechanoregulation at arterial regions of atherosusceptible-SS inflamed by low-dose TNFα.

## Introduction

Atherosclerosis is an inflammatory disease of arteries that develops as early as childhood and underlies the leading causes of death and disability worldwide^1^. It is exacerbated by a complex constellation of local and systemic factors that include proinflammatory cytokines, ApoB-containing serum lipoproteins, advanced glycation end products, bacterial products and viruses, each of which can contribute to endothelial dysfunction^2, 3^. Due to non-uniform vessel geometry, the frictional drag force of flowing blood gives rise to local variations in wall shear stress (SS) that are sensed by endothelial cells (EC) to affect their inflammatory phenotype^4, 5^. At relatively high SS (>10 dynes/cm^2^) this is manifest by EC alignment along the direction of flow that is organized by actin stress fibers, microtubules, and focal adhesions^6^. It is hypothesized that mechanotransduction of atheroprotective signals maintains vascular homeostasis by suppressing inflammation and inhibiting cell proliferation, thrombosis, and increased permeability at EC junctions^6, 7^. Conversely, lipid-rich plaques preferentially develop at focal regions of the arterial wall at so-called atherosusceptible sites, such as within the inner curvature of the aortic arch and at bifurcations. Disturbed flow in these regions gives rise to low magnitude SS (<2 dynes/cm^2^) and steep gradients. Further, at these sites EC are primed for an enhanced response to cytokine stimulation that includes membrane upregulation of cell adhesion molecules (CAM) and increased monocyte recruitment from the circulation^6, 7^. Focal plaque formation at atherosusceptible arterial regions is observed in animal models of atherosclerosis and by postmortem pathohistology of human arteries^4, 8, 9^. Employing a monolayer of human aortic endothelium, we have reported that atheroprotective SS can downregulate the effects of systemic factors on the endothelial response to TNFα stimulation^10, 11^. How EC sense and mechanotransduce signals in proportion to the gradient in SS and modulate inflammatory responses remains ill-defined.

Among the risk factors for atherogenic cardiovascular disease are hyperlipidemia, obesity, and diabetes, which are associated with metabolic dysregulation and an elevated systemic inflammatory state. Endoplasmic reticulum (ER) stress is characterized by an overload of protein processing and is reflected by changes in morphology, specifically an increase in ER-membrane expansion to accommodate the unfolded protein response (UPR)^12, 13^. Activation of the UPR is initiated by dissociation of binding immunoglobulin protein (BiP) from three proximal signaling proteins: activating transcription factor 6 (ATF6), inositol-requiring enzyme 1α (IRE1α), and protein kinase-like extracellular signal-regulated kinase (PERK), which in turn activate distinct downstream effectors to maintain homeostasis. ATF6 is further processed and activated by the Golgi and IRE1α is autophosphorylated leading to upregulation of the transcription factor x-box binding protein 1 (XBP1). PERK phosphorylation leads to activation of eukaryotic initiation factor-2α (eIF2α). Under chronic activation, as occurs in metabolic syndrome, the UPR can regulate autophagy, which under certain conditions can lead to cell apoptosis^14, 15^. ER stress characterized by activation of ATF6 and XBP1 is detected at atherosusceptible regions of disturbed flow in swine fed a normal diet, even in the absence of discernable lesions^14^. Further, studies employing exposure of cultured endothelium to disturbed flow profiles revealed activation of XBP1, BiP, and ATF6, when compared to undisturbed flow or laminar SS^16^. In ApoE-null mice fed high-fat diets, XBP1 is highly expressed at atherosusceptible sites in the aorta and within atherosclerotic lesions^17^. Small molecule chemical chaperones used to protect against ER stress in cultured cells, such as 4-phenyl butyric acid (4-PBA) and the taurine-conjugated derivative of ursodeoxycholic acid (TUDCA), alleviate obesity-induced ER stress and insulin resistance in leptin-deficient (ob/ob) mice^18^, and reduce atherosclerotic lesion progression in ApoE−/− mice^19^. Thus, ER stress and the UPR link cellular metabolic overload with inflammation^3, 20^. Though these studies implicate ER stress in activation of an atherosusceptible phenotype, it is unknown how sensing of SS links to activation of the UPR to elicit an inflammatory response.

The mechanisms of mechanotransduction by EC involve a number of key molecules expressed on the plasma membrane and connected intracellularly via the cytoskeleton^4, 21^. PECAM-1 is a transmembrane glycoprotein which is localized at the junction between EC and considered a direct transducer of mechanical force. A complex comprised of PECAM-1, VE-cadherin, and VEGFR2 lies upstream of integrin activation and is associated with mechanosignaling at focal sites of atherosusceptibility. For example, PECAM-1 deficient mice lack flow-mediated responses such as upregulation of cell adhesion molecules (CAMs) and atherosclerotic lesion formation in the aortic arch^21^. In fact, PECAM-1 knockdown results in a loss in SS-mediated cyclooxygenase-2 activity and prostaglandin I_2_ release, which is dependent upon phosphatidylinositol 3-kinase-induced activation of α_5_β_1_ integrins^22^. While PECAM-1-mediated mechanotransduction activates signaling pathways that affect endothelial function and homeostasis, the extent to which PECAM-1 signals through the ER to locally regulate atheroprone flow-mediated EC inflammatory responses is unknown.

Promoting atherosclerosis at atherosusceptible sites is membrane upregulation of vascular cell adhesion molecule-1 (VCAM-1), which serves as a counter-receptor for very late antigen (VLA)−4-dependent monocyte recruitment^23, 24^. VCAM-1 expression is stimulated by cytokines such as TNFα, and other circulating inflammatory mediators such as apolipoproteins which are elevated by dyslipidemia and metabolic syndrome^2^. In experiments where atherosusceptible levels of SS (2–4 dynes/cm^2^) are superposed with TNFα, human aortic EC (HAEC) upregulate VCAM-1 expression ~3 fold above that of TNFα stimulation in static culture. In contrast, VCAM-1 transcription and expression are suppressed by exposure to atheroprotective SS exceeding 12 dynes/cm² ^11^. These changes are pathologically relevant in the context of atherogenesis as they correlate directly with the efficiency of monocyte recruitment under SS as assessed in an artery-chip microfluidic assay^10, 25^. We have also reported that triglyceride-rich lipoproteins (TGRL) isolated from the serum of hypertriglyceridemic subjects after a high-fat meal were synergistic with low SS in promoting maximal VCAM-1 expression, which was dependent on ER stress signaling and activation of interferon regulatory factor 1 (IRF-1)^11, 12^. A key question remaining is whether SS-mediated atherosusceptibility also involves ER stress upstream of IRF-1 activation, which accounts for the amplification in endothelial inflammation.

In the current study, flow-mediated signaling along a continuous monolayer of HAEC was examined by exposure to a linear gradient of SS generated in a Hele-Shaw microfluidic channel, while in the presence or absence of low dose stimulation by TNFα. Changes in microtubule polarization and EC alignment within the flow field required PECAM-1 expression, as did SS sensitive inflammatory responses. By quantifying changes in CAM expression and leukocyte recruitment with markers of ER stress, we reveal how inflammatory responses are tuned through an ER-stress pathway that regulates an atherosusceptible endothelial phenotype.

## Methods

### Cell culture and treatment protocol

HAEC (Genlantis, lot #2228 derived from a 21-yr-old female, and lot #7F4409 from a 34-yr-old male) were expanded separately on culture flasks in endothelial growth medium-2 (Lonza) with a 1x antibiotic-antimycotic solution (Invitrogen) and used for experiments between passages 5–7. Since unstimulated HAEC express very low levels of VCAM-1 that are not significantly upregulated by SS, cells were stimulated with TNFα to observe the modulatory effect of SS on inflammation^11^. Each lot was characterized for a consistent dose response to TNFα (R&D Systems) stimulation by flow cytometry. HAEC were treated with 0.3 ng/ml TNFα, corresponding to the EC_50_ for upregulation of VCAM-1 or ICAM-1, in growth media with 10% FBS. To inhibit ER stress, HAEC were pretreated with 4-phenylbutyric acid (4-PBA, 1 mM, Sigma) for 1 hrs, then co-treated with TNFα and SS for the time periods indicated. 0.1% DMSO was used as a vehicle control for 4-PBA.

### Shear flow experiments

HAEC were exposed to fluid shear flow within a microfluidic chamber design inspired by Hele-Shaw two-dimensional stagnation flow theory and fabricated from PDMS using soft photolithography, as previously described^10, 11, 26^. The channel width increases from the inlet to the outlet, generating a linearly decreasing magnitude of SS as a function of axial distance (x) from the inlet and resulting in a stagnation point just proximal to the outlet. For a constant input flow rate, the wall SS (τ_w_) along the centerline of the channel changes linearly with distance down the channel (x) as follows:

$${\tau }_{w}=\frac{6\mu Q}{{h}^{2}{w}_{1}}(1-\frac{{\rm{x}}}{L})$$

where μ is the viscosity of the flow medium, Q is the volumetric flow rate, h is the channel height, w_1_ is the channel width at the inlet, and L is the length of the channel. HAEC were seeded on collagen type-1-coated (100 μg/ml, Invitrogen) glass coverslips and grown to 90–95% confluency. PDMS microfluidic chambers were then reversibly vacuum-adhered to the HAEC monolayers. Leibovitz-15 media (Gibco) supplemented with 10% FBS, endothelial BulletKit (Lonza), and 1X antibiotic-antimycotic was used as flow medium to maintain pH in the absence of CO_2_ regulation. A closed-loop flow system was powered by a Masterflex L/S peristaltic pump (Cole-Parmer) in a humidified chamber heated to 37 °C. To observe changes in CAM expression, HAEC were stimulated at a TNFα concentration corresponding to the EC_50_ for CAM upregulation (0.3 ng/ml) in the presence and absence of SS for 4 hrs., unless otherwise indicated.

### Cell transfection

Lentivirus transduction particles (Sigma) containing shRNA against XBP1 (TRCN000019808), eIF2α (TRCN0000143559), ATF6 (TRCN0000017855), and PECAM-1 (TRCN0000057801) were introduced to HAEC at MOI 2.5 for 12 hrs in media, then diluted to an MOI of 1.25 for another 36 hrs. Puromycin (Sigma) was added to the growth media at 1 µg/ml for 48 hrs to select for transfected cells. The efficiency of knock-down was confirmed via western blot to be 75–80% in each case.

### Immunofluorescence microscopy

Treated cells were rinsed in PBS and fixed in 4% paraformaldehyde (Electron Microscopy Sciences) for 10 min. Samples were blocked in PBS with 20% donkey serum (Invitrogen) and 1% HSA for 20 min, and then stained with the primary antibody anti-Calreticulin (1:200, 6468), anti-α-Tubulin (1:500, Sigma no. T9026), anti-VCAM-1 (1:10, BD Biosciences no. 555647), anti-ICAM (1:20, Biolegend no. 322714), anti-XBP-1 (1:25, 7160), anti-p-eIF2α (1:50, Abcam no. 32157), anti-eIF2α (1:25, 133227), or anti-ATF6 (1:25, 22799) for 2 hrs. All primary antibodies were purchased from Santa Cruz Biotechnology unless otherwise noted. Subsequently, fluorescently conjugated secondary antibodies were applied in blocking buffer to the cells based on their reactivity and emission properties. Primary mouse antibodies were stained with Alexafluor 555 goat anti-mouse (Life Technologies no. A21425); primary goat antibodies were stained with Alexafluor 546 rabbit anti-goat (Life Technologies no. A21085); primary rabbit antibodies were stained with Alexafluor 647 goat anti-rabbit (Life Technologies no. A21245) all diluted to 1:500 in blocking buffer. To detect intercellular proteins 0.1% Triton X-100 (Sigma) was added to blocking buffers and to staining buffers. Immunolabeled samples were mounted on microscope slides using Vectashield with DAPI (Vector Laboratories). Using a Nikon inverted microscope equipped with a 16-bit Zyla sCMOS (Andor) or an Electron Multiplying C9100 (Hamamatsu) camera and a 60x objective (N.A. 1.49, Nikon), five representative images were captured using 4 × 4 binning (2.33 pixels per μm resolution) at each SS in each sample. Multiple images corresponding to a fixed axial position *x* were acquired such that they deviated from the expected SS by less than 0.2 dynes/cm². Mean fluorescence intensity of >60 individual cells per SS magnitude per sample was quantified using ImageJ (National Institutes of Health). The trend in CAM regulation captured in large image stitches was measured using seven centerline intensity profiles (5000 points sampled), filtered by median intensity, and plotted versus shear stress using a scatter plot. The Lowess curve fitting algorithm was applied to the data in the scatter plots.

### Microtubule alignment

To induce cell alignment, HAEC were sheared for 24 hrs, fixed with paraformaldehyde, and immunolabeled for α-tubulin. Microtubule alignment was quantified as a readout of cell morphology, using the ImageJ plugin *Directionality*. A power analysis, extracted from the fast Fourier transform of representative images acquired at each SS, reported on the relative alignment with the flow field (Supplementary Fig. 1). In this analysis, a peak in power centered at an angle of 0 degrees indicated the greater polarity of alignment in the direction of flow, while random orientation was associated with relatively constant power over all angles. Calculating the area under the curve for discrete SS values provided a metric by which to compare to the static case.

### ER morphology

HAEC expressing and lacking the mechanosensitive protein PECAM-1 were exposed to TNFα and SS for 4 hrs, immunolabeled for the ER resident protein calreticulin, and imaged by confocal laser scanning microscopy (Olympus FV1000). ER expansion (associated with a state of ER stress) was measured by calculating the coefficient of variation (CV) of cytoplasmic calreticulin pixel intensity, which increases with increasing spatial heterogeneity.

### Leukocyte adhesion assays

Human subjects were recruited according to Institutional Review Board-approved protocols at the University of California, Davis under informed consent. Mononuclear cells (MNC) and polymorphonuclear neutrophils (PMN) were isolated from human blood using Lymphoprep density gradient (Stemcell Technologies), and Polymorphprep (Sigma), respectively. PMN were stained with DiD (Thermo Fisher) and combined with unstained MNC so that each was suspended at 2 × 10^6^ cells/ml in HBSS with calcium and magnesium. Following treatment under SS as described, the Hele-Shaw device was carefully removed to retain intact HAEC monolayers, and replaced with a second PDMS microfluidic design, consisting of straight channels running parallel to the axis of flow. A syringe pump was used to draw the MNC and PMN into the chamber at a constant flow rate corresponding to a SS of 2 dynes/cm^2^. After 10 min of flow, the HAEC were rinsed with HBSS, and adherent MNC and PMN (defined as moving less than 0.5 cell diameters in 10 sec.) were enumerated under SS via bright-field and fluorescent microscopy. A 20x objective was used to capture 3 images per SS magnitude per sample.

### Data analysis

Data were analyzed using GraphPad Prism (version 5.02) software. ANOVA was used to compare multiple treatments and the Newman-Keuls posttest to identify differences. Repeated measures ANOVA and Dunnett’s posttest was used to compare multiple shear stresses to the static control within an experimental group. Student’s t-test was used to compare 2 experimental groups at a given shear stress. Two-tailed *P* values of < 0.05 were considered statistically significant unless otherwise indicated.

## Results

### HAEC are mechanoresponsive to inflammatory stimulation along a linear gradient of hydrodynamic shear stress

**Figure 1:**
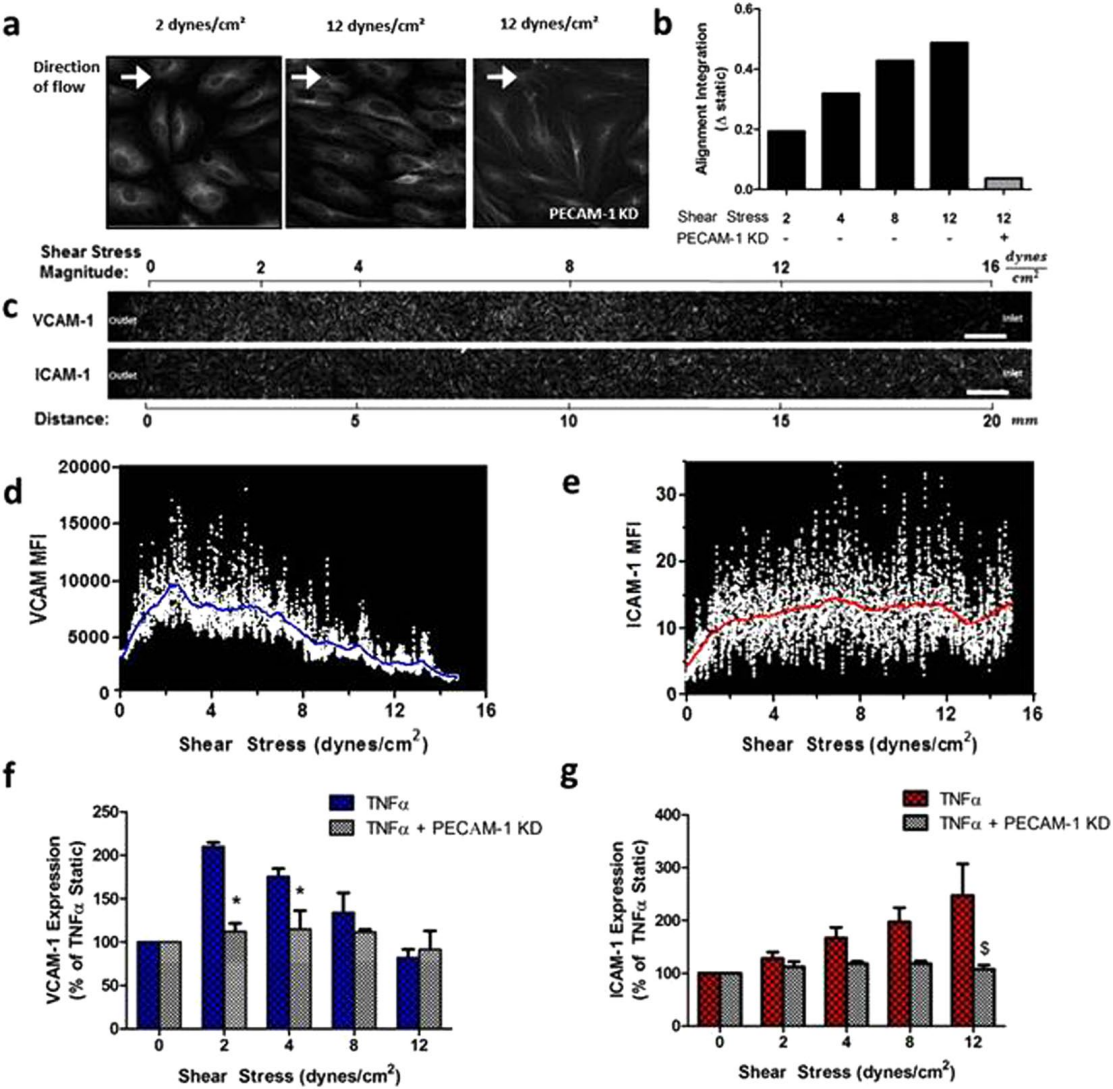
HAEC exhibit differences in morphology and inflammatory phenotype in a hydrodynamic SS gradient. Monolayers were exposed to TNFα and a fluid SS gradient (0–16 dynes/cm^2^ over 20 mm) in a microfluidic device. PECAM-1 knock-down cells were used as a mechano-insensitive control. Microtubules (MT) were immunolabeled, and fiber alignment was quantified as a readout of cell morphology at 24 hrs. (**a**) Representative images stained for MT. (**b**) Change in alignment from the static condition, calculated from the integral of the FFT power analysis. HAEC monolayers were treated with TNFα and SS as above for 4 hrs and labeled for VCAM-1 and ICAM-1 expression. (**c**) Representative large image stitch spanning the centerline of the chamber (Scale bar = 1 mm), and (**d**,**e**) Centerline intensity profile vs. SS with Lowess fit for VCAM-1 and ICAM-1 within the same monolayer. (**f**,**g**) Expression profile for VCAM-1 and ICAM-1 in HAEC treated as above, or also transfected with shRNA against PECAM-1 or a scrambled control. Values are mean ± SE, n = 3–5, *P < 0.05 vs same SS, ^$^P = 0.057 vs same SS.

The Hele-Shaw flow chamber design enables the direct visualization of SS-dependent regulation of the cell and molecular events that follow inflammatory stimulation of endothelium within a confluent monolayer exhibiting tight junctions. The flow rate was set to produce a linear gradient in SS spanning a physiological range in arteries from 16 dynes/cm^2^ (atheroprotective) to near zero (atherosusceptible) over a distance of 20 mm that corresponds to ~800 EC diameters along a monolayer spanning the channel. In response to 24 hrs of continuous laminar shear, microtubule polarization correlated directly with the alignment of HAEC along the flow axis. Maximum alignment occurred in the vicinity of high SS near the channel inlet and decreased proportionally down the flow field (Fig. 1a,b). HAEC adopted a fusiform morphology at and above 12 dynes/cm^2^, which was associated with the maximal orientation of microtubules (Supplementary Fig. 1). At progressively lower SS, microtubule orientation decreased in a linear manner toward a minimum at the 0 dynes/cm^2^ stagnation point, where EC were randomly orientated. In contrast, microtubule intensity along with EC density, as assessed by DAPI positive nuclei per substrate area, remained constant along the monolayer from inlet to outlet (Supplementary Fig. 2).

We next examined the response of HAEC sheared while stimulating with TNFα at 0.3 ng/ml, the EC_50_ for CAM upregulation^27^. VCAM-1 and ICAM-1 immunofluorescence was quantified along the microfluidic channel, depicted as a photomontage (Fig. 1c). Mean fluorescence was quantified by digitizing the centerline fluorescence intensity profile over 1000 discrete slices at 20 µm wide intervals along the channel (1d, 1e). HAEC VCAM-1 expression at the stagnation point within the Hele Shaw increased by 4-fold that of unstimulated controls. This level of up-regulation was equivalent to HAEC cultured under static conditions in tissue culture dishes (Supplementary Fig. 3). TNFα-stimulated VCAM-1 expression increased steeply along the channel, rising from the static control baseline to reach a peak ~1.5-fold above this level at 2 dynes/cm^2^, which corresponded to 2.5 mm along the channel (Fig. 1f). From peak VCAM-1, the expression decreased gradually to below baseline expression at 12 dynes/cm^2^ near the channel inlet. In contrast, ICAM-1 expression rose more gradually over the shear gradient, reaching ~1.5-fold of static baseline level above 12 dynes/cm^2^ (Fig. 1g). This pattern of CAM mechanoregulation is consistent with previous reports^10, 11^. To demonstrate the role of mechanotransduction in mediating this response to SS, HAEC were pretreated with shRNA to knock-down the expression of PECAM-1, a membrane receptor which contributes to mechanosensing at EC junctions^21, 28^. PECAM-1 deficient cells maintained the increase in VCAM-1 and ICAM-1 expression in response to TNFα stimulation under static conditions and maintained the capacity to form a confluent monolayer under fluid SS. However, PECAM-1 knockdown abrogated cellular alignment in response to high SS, reflected in the lack of microtubule polarization, which was comparable to the static condition (Fig. 1a,b). Knockdown of PECAM-1 abrogated the shear-dependent modulation of CAM expression with TNFα stimulation along the SS gradient. In contrast, a scrambled control shRNA retained the characteristic increase to peak VCAM-1 expression at 2 dynes/cm^2^ and ICAM-1 at 12 dynes/cm^2^ (Fig. 1f,g). These data confirm the presence of a mechanosignaling pathway underlying the changes in CAM expression observed along the SS gradient.

### ER expansion is regulated by SS in a PECAM-1 dependent manner

**Figure 2:**
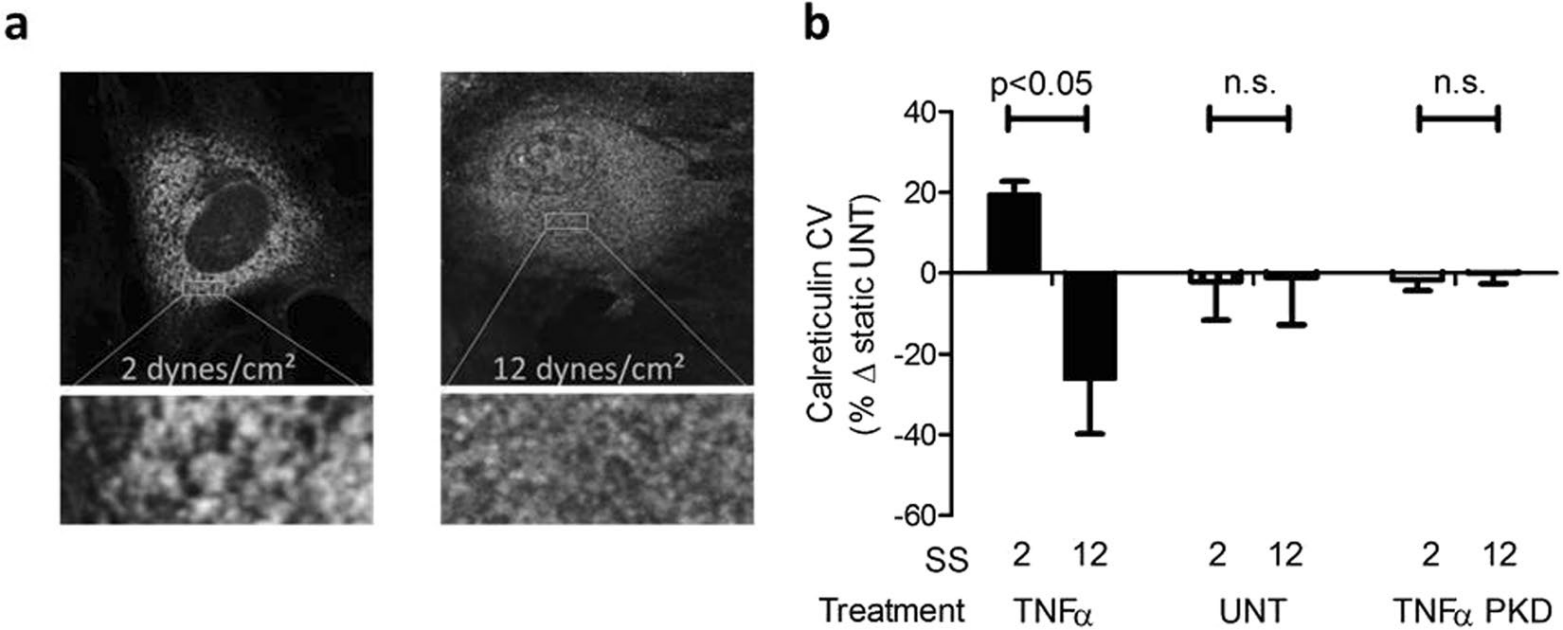
ER expansion is altered by SS in a PECAM-1 dependent manner. HAEC expressing and lacking the mechanosensitive protein PECAM-1 were exposed to TNFα (0.3 ng/ml) and SS for 4 hrs and immunolabeled for the ER resident protein calreticulin. (**a**) Representative confocal images from low (2 dynes/cm^2^) and high (12 dynes/cm^2^) SS regions in TNFα-stimulated cells. (**b**) Coefficient of variation (CV) of calreticulin pixel intensity, a measure of ER expansion associated with ER stress. Values are mean ± SE, n = 3–5.

To determine the extent to which hydrodynamic and ER stress are linked in regulating the inflammatory response in endothelium, we labeled the ER membrane with a fluorescent antibody for calreticulin and measured changes in ER morphology, which is strongly correlated with function^12, 13^. ER expansion was not significantly altered in response to SS alone in the absence of TNFα. Stimulation with TNFα induced ER expansion, evidenced by an increase in the spatial heterogeneity of calreticulin staining by 17% over static levels at 2 dynes/cm^2^, which dropped steadily to 26% below static as SS increased to 12 dynes/cm^2^ (Fig. 2). Modulation of ER expansion by SS was not observed in PECAM-1 deficient HAEC or unstimulated static HAEC. These data confirm a pattern of ER stress that is dependent upon cytokine stimulation and mechanosensing along the gradient of SS.

### SS-modulation of TNFα-induced VCAM-1 expression is ER stress-dependent

**Figure 3:**
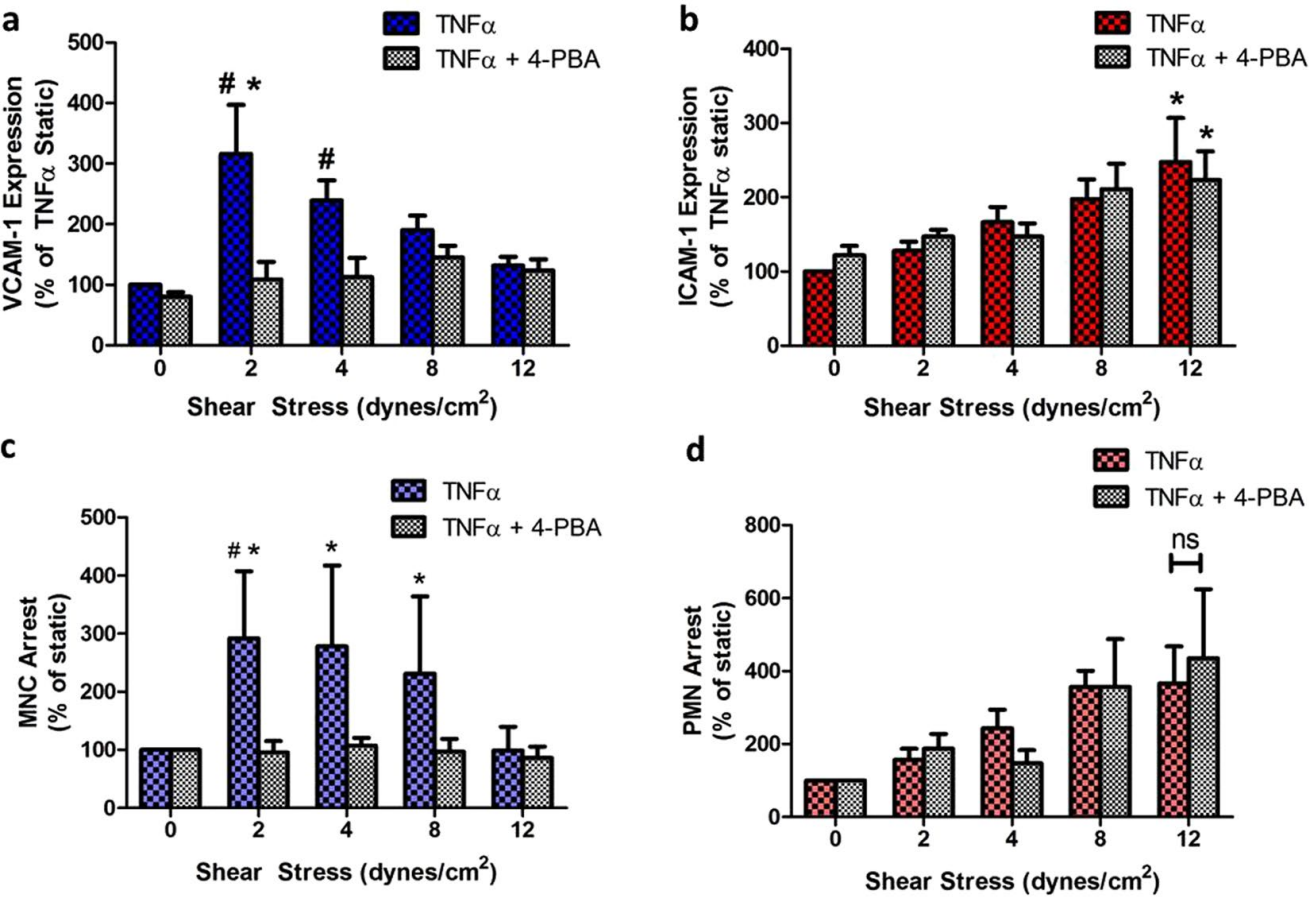
SS-modulation of TNFα-induced VCAM-1 expression is ER stress-dependent. HAEC were exposed to TNFα and SS for 4 hrs in the presence or absence of an inhibitor of ER stress, 4-PBA, and immunolabeled for (**a**) VCAM-1 or (**b**) ICAM-1. Isolated (**c**) mononuclear cells (MNC) and (**d**) polymorphonuclear neutrophils (PMN) were flowed over preconditioned HAEC and firmly arresting cells were enumerated. Values are mean ± SE, n = 3–5, ^#^P < 0.05 vs static TNFα. *P < 0.05 vs same SS.

Since CAM upregulation is an early event in atherogenesis, we next examined the role of ER stress in the regulation of VCAM-1 and ICAM-1 expression along the channel. HAEC were treated with the ER stress inhibitor 4-PBA, stimulated with TNFα, and exposed to shear flow. The rise in VCAM-1 expression at low magnitude SS was abrogated in the presence of the inhibitor, effectively maintaining cytokine-stimulated expression at a constant level equivalent to the static condition throughout the SS gradient (Fig. 3a). In contrast, inhibition of ER stress did not alter the upregulation of ICAM-1 at any SS (Fig. 3b). To examine the role of ER stress in CAM-mediated leukocyte recruitment, isolated human mononuclear cells and neutrophils were separately perfused over HAEC monolayers that had previously been stimulated with TNFα and sheared in the Hele-Shaw channel. A straight channel was positioned over the HAEC preconditioned within the SS gradient to flow leukocytes at a constant SS of 2 dynes/cm^2^. MNC captured and rolled at all positions along the channel and transitioned to shear resistant arrest with the greatest efficiency at a position that correlated with the highest level of SS-modulated VCAM-1 expression. MNC arrest was completely blocked by treatment with VCAM-1 or VLA-4 function-blocking antibodies (data not shown)^11^. Moreover, inhibition of ER stress by pretreatment with 4-PBA inhibited MNC recruitment down to static levels along the channel, commensurate with the reduction in VCAM-1 expression (Fig. 3c). Neutrophils, which lack functional levels of VLA-4 but express LFA-1 (CD11a/CD18) receptors, rolled over preconditioned inflamed HAEC and PMN adhesion correlated directly with the steady increase in ICAM-1 expression (i.e. the ligand for LFA-1) down the channel. This pattern of PMN arrest was not affected by 4-PBA inhibition (Fig. 3d). Together, these data indicate that ER stress is involved in the SS regulation of VCAM-1 expression and in modulating the efficiency of monocyte recruitment, but not the regulation of ICAM-1-dependent PMN arrest.

### Activation of XBP1 and eIF2α arms of the UPR is necessary for SS regulation of VCAM-1

**Figure 4:**
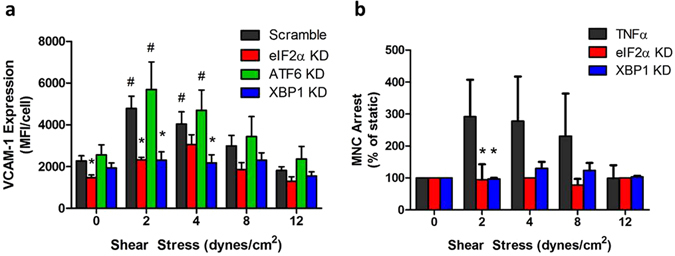
XBP1 and eIF2α activation are necessary for SS regulation of VCAM-1. (**a**) HAEC were transfected with shRNA against eIF2α, ATF6, XBP1, or a scrambled control. Monolayers were treated with SS and TNFα for 4 hrs then analyzed for VCAM-1 protein expression via immunofluorescence microscopy. (**b**) MNC arrest was quantified in HAEC lacking XBP1 or eIF2α. Values are mean ± SE, n = 4–5, ^#^P < 0.05 vs static within group. *P < 0.05 vs scrambled control at respective SS.

To elucidate which component of the UPR plays a role in the regulation of SS-mediated VCAM-1 expression and monocyte arrest, HAEC were transfected with shRNA against eIF2α, ATF6, or XBP1, to knockdown primary signaling proteins associated with activation of the 3 arms of the UPR. HAEC monolayers were pretreated with each shRNA or scrambled control shRNA, cytokine-stimulated and sheared in the Hele-Shaw channel, followed by quantitation of VCAM-1 expression and MNC arrest. Knockdown of eIF2α or XBP1, but not ATF6, abolished the SS-dependent upregulation of VCAM-1 at 2 dynes/cm^2^, and efficiently suppressed VCAM-1 expression across the SS gradient to static levels (Fig. 4a). In contrast to XBP1 inhibition, eIF2α knockdown also inhibited VCAM-1 upregulation by ~20% of the static level, revealing a non-SS-mediated role in inflammatory signaling via TNFα activation. Consistent with the inhibition of VCAM-1, knockdown of XBP1 or eIF2α inhibited the pattern of increased MNC arrest detected at atherosusceptible low SS on inflamed HAEC (Fig. 4b).

### SS differentially modulates activation of ER stress response pathways

**Figure 5:**
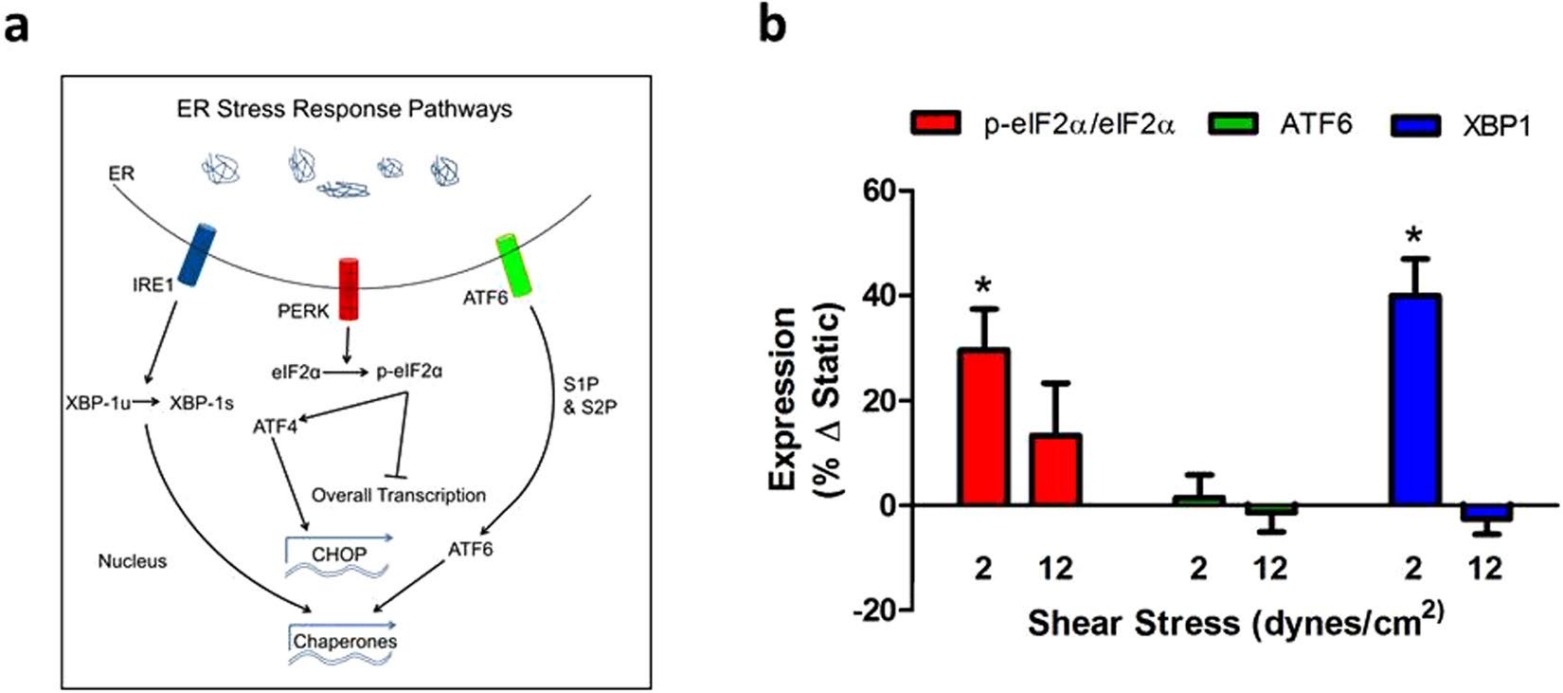
SS differentially modulates activation of ER stress response pathways. (**a**) ER stress is characterized by overload of protein processing and is reflected by changes in morphology, specifically an increase in ER-membrane expansion to accommodate the unfolded protein response (UPR)^12, 13^. Activation of the UPR is initiated by dissociation of binding immunoglobulin protein (BiP) from three proximal signaling proteins: activating transcription factor 6 (ATF6), inositol-requiring enzyme 1α (IRE1α), and protein kinase-like extracellular signal-regulated kinase (PERK), which in turn activate distinct downstream effectors in order to maintain homeostasis. ATF6 is further processed and activated by the Golgi and IRE1α is autophosphorylated leading to upregulation of the transcription factor x-box binding protein 1 (XBP1). PERK phosphorylation leads to activation of eukaryotic initiation factor-2α (eIF2α). (**b**) HAEC were treated with TNFα and SS for 4 hrs and immunolabeled for the ER stress pathways proteins eIF2α, p-eIF2α, ATF6, XBP1. Values are mean ± SE, n = 3–4, *P < 0.05 comparing low to high SS within group.

We next directly probed the canonical UPR pathways by quantifying immunofluorescence to determine the extent to which each arm is activated by SS and contributes to VCAM-1 upregulation. HAEC were stimulated with TNFα in the Hele-Shaw channel for 2 hrs, permeabilized, and immunolabeled to quantify the relative expression of XBP1, ATF6, and phospho-eIF2α/total-eIF2α (Fig. 5). Differential activation of ER stress along the SS gradient was evident when compared to spatial regulation of VCAM-1 expression. Specifically, at 2 dynes/cm^2^, corresponding to maximal upregulation of VCAM-1, eIF2α and XBP1 activity were enhanced by 27% and 40% above static, respectively. At a relatively high SS of 12 dynes/cm^2^, corresponding to low VCAM-1 expression, p-eIF2α, and XBP1 activity were suppressed. In contrast, significant changes in ATF6 were not observed along the SS gradient. We conclude that VCAM-1 expression is mechanoregulated through activation of the eIF2α and XBP1 arms of the UPR at atherosusceptible low SS.

### Shear regulation of IRF-1 activation is signaled by ER stress

**Figure 6:**
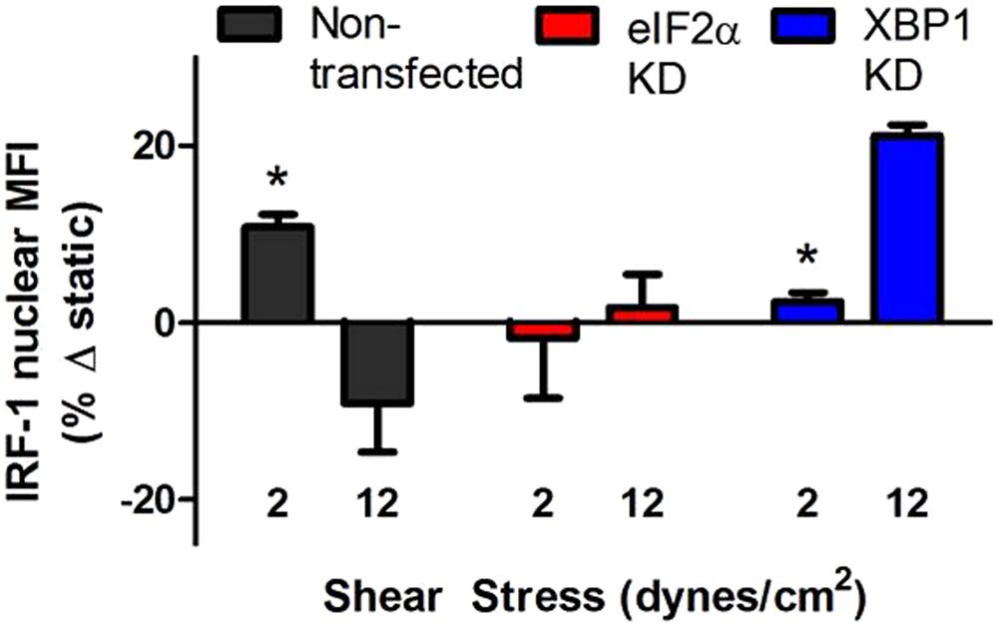
Shear regulation of IRF-1 activation is signaled by ER stress. HAEC transfected with shRNA against eIF2α or XBP1 were treated with TNFα and SS for 2 hrs and nuclear IRF-1 was quantified by immunofluorescence microscopy and compared to control. Values are mean ± SE, n = 3–4, *P < 0.05 comparing low to high SS within group.

The transcription factor IRF-1 is one of the several regulatory elements that specifically promotes expression of VCAM-1 following inflammatory activation of EC^29^. It is expressed at low levels in the cytosol of unstimulated HAEC and under SS in the Hele Shaw channel. TNFα stimulation elicits its upregulation and recruitment to the nucleus at peak levels within the region of atherosusceptible SS^11^. The increase in nuclear translocation of IRF-1 accounts for the amplification in VCAM-1 expression in HAEC exposed to atherosusceptible low SS^11^. Here we examined the mechanoregulation of IRF-1 translocation by ER stress. Consistent with the relative changes in VCAM-1 expression, activated IRF-1 increased over static by 11% at low SS and dropped ~10% below static at high SS (Fig. 6). Peak IRF-1 expression at low SS was inhibited in the presence of shRNA targeting either eIF2α or XBP1. At high atheroprotective levels of SS, eIF2α knockdown reversed the reduction in IRF-1, while XBP-1 knockdown significantly enhanced levels above static expression. We conclude that SS signaling leads to activation of 2 arms of the UPR that differentially regulate IRF-1 activity and VCAM-1 expression.

## Discussion

**Figure 7:**
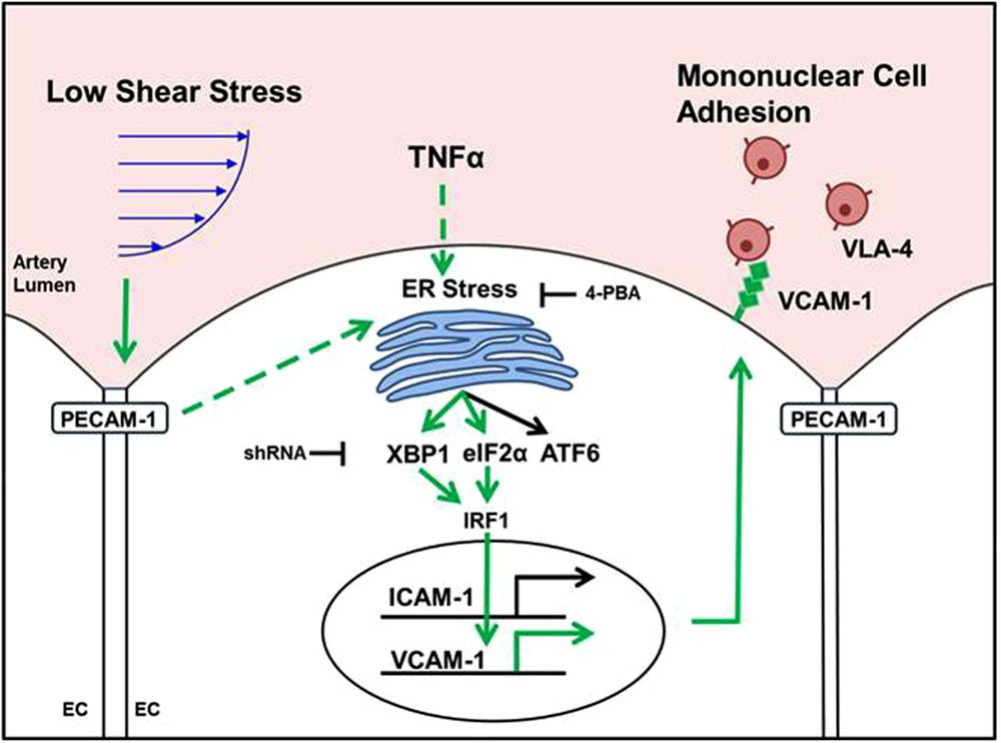
Schematic of a mechanosensitive pathway whereby low magnitude SS acts through the ER stress response pathways XBP1 and eIF2α to promote IRF-1-mediated VCAM-1 expression and VLA-4-dependent monocyte adhesion.

The sensing of mechanical cues imparted by hemodynamics constitutes a fundamental means by which the endothelium participates in the maintenance of vascular homeostasis, including the focal regulation of inflammatory responses that contribute to atherogenesis. Low magnitude time-averaged SS and steep SS gradients are characteristics of sites susceptible to atherosclerosis and are accompanied by changes in EC morphology and inflammatory phenotype that are distinct from regions of high atheroprotective SS^4, 7^. Employing a Hele-Shaw microfluidic channel, we applied high-resolution fluorescence imaging to produce a detailed spatial map of inflammatory signaling as a function of SS that varied from anti- to pro-atherogenic along a continuous monolayer of HAEC. Changes in microtubule polarization and EC alignment along the flow field were dependent upon PECAM-1-mediated mechanotransduction of SS as depicted in the summary schematic of Fig. 7. Cytokine-stimulated VCAM-1 expression peaked at atherosusceptible SS, consistent with previous reports^11^, and was also dependent on PECAM-1 function. ER expansion attributed to XBP1 and eIF2α activity varied inversely with the magnitude of SS, peaking at atherosusceptible levels that correlated with the highest amplification in IRF-1 activation, maximal VCAM-1 expression, and preferential recruitment of monocytes under flow. Inhibition of ER stress or knockdown of XBP1 or eIF2α abrogated the SS-dependent modulation of VCAM-1, while ICAM-1 expression and PMN recruitment were unaffected. These data reveal a central role for ER stress in the focal hemodynamic regulation of cytokine-induced VCAM-1 expression and monocyte recruitment, early inflammatory events in atherosclerosis.

*Artery-on-a-chip* technology, as applied here, reveals how hydrodynamic SS spatially and temporally regulates endothelial inflammatory responses. A previous study documented the utility of this device for detecting changes in VCAM-1 expression with high spatial acuity in response to increments in SS on the order of 0.25 dynes/cm^2^ ^10^. It was concluded that HAEC sense the relative magnitude of SS rather than the steepness of the gradient^10^. Consistent with this is the current finding that VCAM-1 effectively doubled in expression between the peak at 2 dynes/cm^2^ and static SS at the channel outlet, a distance corresponding to 2.5 mm or ~100 EC lengths. Over this same distance, ER expansion increased by 17%, and this corresponded to boosts in the expression of 27% in eIF2α, 40% in XBP1, and 11% in IRF-1. These ER stress markers gradually decreased along the channel as shear stress increased to 12 dynes/cm^2^. As summarized in Fig. 7, fluid SS is sensed in a PECAM-1-dependent manner and, through a mechanism still incompletely understood, converges with cytokine signaling to affect ER stress and activation of the UPR. This sequence of molecular events culminates in changes in VCAM-1 transcription and translation in proportion to SS magnitude that are of pathophysiological significance as evidenced by accompanying changes in recruitment efficiency of MNC.

In pre-lesional animal models of atherosclerosis, activation of the UPR occurs at disturbed flow regions. This implicates a role for arterial hemodynamics in initiation of the ER stress response, which superposes with other metabolic drivers of inflammation to confer an atherogenic bias^14, 16, 30^. Here we examined the three arms of the UPR for their contribution to shear modulated VCAM-1 expression and MNC recruitment in response to an acute inflammatory challenge, as these represent early changes during atherogenesis. In contrast to previous studies that reported sustained UPR activation in cultured endothelium subjected to atherosusceptible flow in the absence of TNFα^16^, in our study exposure to low SS for up to 24 hrs did not induce activation of the UPR without TNFα stimulation (Supplementary Fig. 4). These differences may in part be attributed to experimental parameters, including our use of HAEC harvested from healthy donors and maintained in a low activation state in cultured unstimulated monolayers as indicated by low constitutive VCAM-1 expression that is not significantly upregulated by SS in the absence of cytokine stimulation^11, 12^. Consistent with our previous findings that ER stress plays a relatively modest role in TNFα-induced upregulation of VCAM-1 on HAEC under static conditions, here we detected ~30% inhibition at 0 dynes/cm^2^ in the presence of 4-PBA (Fig. 3a)^12^. Specific knock-down revealed this TNFα contribution in static HAEC was signaled via eIF2α activity (Fig. 4a). However, in cells simultaneously sheared and stimulated with TNFα, we observed differential activation of XBP1 and eIF2α in response to low SS compared to high SS ﻿(Supplementary Fig. 5). Discrete knock-down of XBP1 and eIF2α using lentiviral-delivered shRNA confirmed that each factored prominently into the cytokine-induced VCAM-1 response and MNC arrest under low SS. These results are consistent with ER stress promoting an atherosusceptible phenotype at sites of disturbed flow in arteries.

TNFα is elevated in the serum of individuals exhibiting cardiometabolic risk factors and expressed locally within atherosclerotic lesions^31^. Its ligation stimulates a potent increase in VCAM-1, ICAM-1, and E-Selectin, that is further modulated up or down by exposure to hydrodynamic stress^10^. The induction of CAMs by pro-inflammatory cytokines occurs in large part through activation of the transcription factors NFκB and AP-1. However, the VCAM-1 promoter contains a unique regulatory element compared to other endothelial CAMs, in that the transcription factor IRF-1 acts cooperatively with NFκB to induce a maximal response to TNFα^32^. We previously reported that the boost in VCAM-1 expression induced by superposition of low SS and TNFα was not due to enhanced activity of NFκB or AP-1, but was dependent on IRF-1 activity^11^. By silencing IRF-1 activity, VCAM-1 expression was reduced to a level equivalent to stimulation under static conditions over the range of SS^11^. Consistent with those findings, we observed here that suppression of XBP1 or eIF2α abrogated the increase in VCAM-1 expression mediated by IRF-1 upregulation at atherosusceptible SS. UPR-mediated regulation of VCAM-1 was less consistent at high SS, where XBP-1 knockdown led to an unexpected increase in IRF-1. Others have demonstrated that transcription factors such as KLF2 and certain micro-RNAs are responsible for regulation of inflammatory responses at high SS, including the suppression of TNFα-stimulated VCAM-1^8, 30^. Together with our data, this points to an ER stress-independent mechanism within atheroprotective regimes of SS. In contrast, the up-regulation of ICAM-1 by high magnitude SS is dependent on the activation of NFκB and insensitive to changes in KLF2^7, 33, 34^. We conclude that KLF2 and factors unaffected by ER stress account for VCAM-1 suppression and ICAM-1 upregulation by atheroprotective high levels of SS.

There is limited published data on how hydrodynamic forces are mechanotransduced into biochemical signals that influence ER stress. One study reported that BiP upregulation by atherosusceptible SS in a cell culture model was dependent upon α_2_β_1_ integrin ligation^16^. The prominent role for PECAM-1 in mechanotransduction through pathways that involve cross-talk with integrins motivated our examination of its role in the shear-mediated regulation of VCAM-1^21, 22^. PECAM-1 knockdown resulted in the loss of flow-mediated cell alignment, microtubule polarization, ER expansion, and the mechanotransduced regulation of VCAM-1 and ICAM-1. Additional studies are necessary to elucidate the downstream signaling molecules that link PECAM-1 activity to ER stress responses. ER morphology is closely related to the cell’s protein maturation and secretion capabilities. For example, expansion of ER membrane surface area generates additional volume to process nascent proteins. It is generally accepted that ER expansion and biogenesis are responses that feed back to alleviate a state of ER stress^35^. ER expansion is a dynamic process that is impacted not only by the different arms of UPR, but other factors including lipid droplet interaction, lipid homeostasis, as well as interactions with the mitochondrial and plasma membrane junctions^35^. It is also possible that SS acts directly on microtubules and their close association with ER to affect morphology upstream of the UPR. For example, microtubules interact with the ER through microtubule-associated proteins (EB1) and molecular motors (kinesin-1, dynein) that bind proteins on the ER membrane (e.g., STIM1) which may drive ER membrane expansion^35^. Real-time high-resolution confocal imaging of microtubule dynamics under defined hydrodynamic shear as it relates to ER expansion could reveal these structure-function relationships. Preliminary data from our laboratory indicates that uncoupling microtubules from the ER with griseofulvin results in abrogation of the shear-mediated VCAM-1 response (data not shown). Further characterization of the spatial and temporal responses mediated by the ER and how these contribute to mechanosignaling may reveal the focal origins of arterial disease.

It is noteworthy that exposure to SS alone did not significantly upregulate the UPR, but in the context of acute TNFα stimulation, low SS greatly enhanced ER stress and activation of the UPR. An experimental limitation is that application of shear to a HAEC monolayer in the Hele-Shaw channel does not fully recapitulate the complexity of disturbed flow in an artery. Specifically, flow reversal or oscillation may factor prominently into promoting an atherosusceptible phenotype, especially under conditions where cells are chronically exposed to shear in culture or *in vivo*. We previously reported that pulsatile flow (12 ± 5 dynes/cm^2^) or oscillatory flow (2 ± 5 dynes/cm^2^) yielded equivalent changes in VCAM-1 expression in response to TNFα stimulation compared to steady SS of the same time-averaged magnitude (Supplementary Fig. 6). Moreover, our model is an acute exposure to SS, whereas atherosclerosis is a chronic disease that develops over decades of exposure to acute repetitive insults, such as may be elicited by a diet high in saturated fat^36^. The postprandial state is associated with a pro-inflammatory milieu, which includes elevated circulating levels of cytokines and atherogenic lipoproteins in the serum. In cultured HAEC, a peak in VCAM-1 expression occurs within 4 hrs of stimulation with TNFα. Preconditioning HAEC to SS for 20 hrs prior to TNFα stimulation did not augment VCAM-1 upregulation, suggesting that acute SS is sufficient to prime the response to TNFα^11^. Additional factors also come into play during atherogenesis including the presence of smooth muscle cells and resident immune cell activity in the arterial intima, which are absent in the microfluidic channels.

Despite these limitations, the Hele-Shaw-based artery-chip provides a means of dissecting *ex vivo* the respective roles of SS magnitude and other mediators of inflammation, such as cytokines and atherogenic lipoproteins, to the intracellular signaling pathways that drive endothelial inflammatory phenotype and promote recruitment of monocytes associated with atherogenesis. Here we directly link SS-mediated mechanotransduction with an ER-dependent inflammatory response. We conclude that ER stress plays a prominent role in VCAM-1 upregulation by atherosusceptible low SS in TNFα-activated HAEC.

## Electronic supplementary material

Supplementary Data
