# Supplementary material for: Atherosusceptible Shear Stress Activates Endoplasmic Reticulum Stress to Promote Endothelial Inflammation
Source: Sci Rep. 2017 Aug 15;7:8196. doi: 10.1038/s41598-017-08417-9 (PMC5557756; doi:10.1038/s41598-017-08417-9)
Supplement: Supplementary file 1 — Supplementary Data [file 41598_2017_8417_MOESM1_ESM.pdf]

Atherosusceptible Shear Stress Activates Endoplasmic Reticulum Stress to Promote Endothelial Inflammation

Authors:
Keith A. Bailey[1], Fawaz G. Haj PhD[2,3], Scott I. Simon PhD[1], Anthony G. Passerini PhD[1,*]

[1] Department of Biomedical Engineering, [2] Department of Nutrition, [3] Department of Internal Medicine, University of California, Davis, CA

*Corresponding Author:
Anthony G. Passerini, PhD
Department of Biomedical Engineering
University of California, Davis
451 Health Sciences Dr., Davis, CA 95616
Phone: 530-754-6715
email: agpasserini@ucdavis.edu

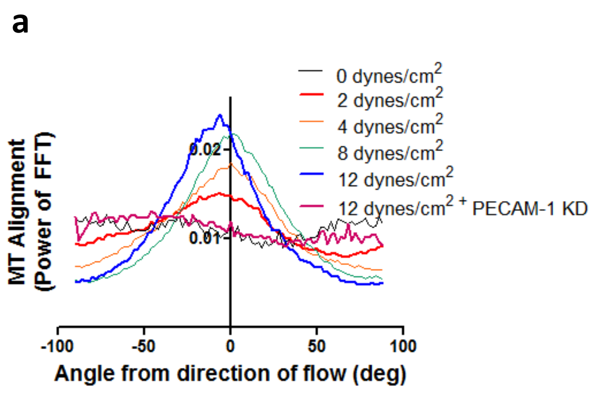

Supplement Figure 1 HAEC were exposed to a fluid shear stress gradient within a microfluidic device. (**a**) To examine morphology at 24 hrs, HAEC were immunolabeled for microtubules (MT), and analyzed for fiber alignment as a readout of cell morphology using the ImageJ plugin Directionality. This program analyzes the power of the fast Fourier transform (FFT), which reports on the periodicity with respect to angle from direction of flow. PECAM-1 knockdown cells were used as a mechano-insensitive control. (**b**) To compare alignment at different SS conditions, integration of the curves was plotted as a function of SS as presented in Fig 1b.

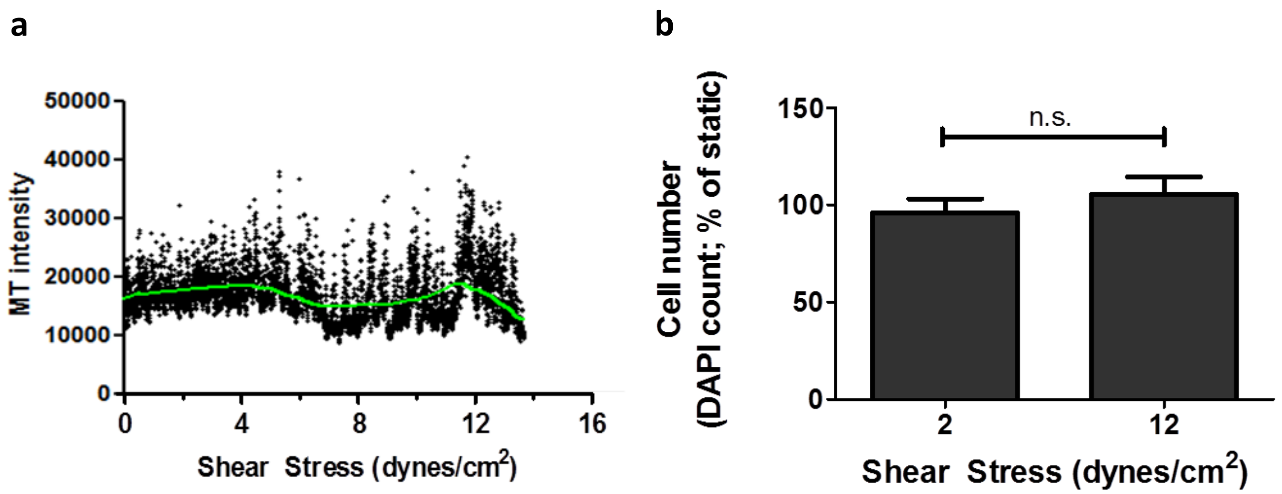

Supplement Figure 2: Microtubule intensity and cell number do not vary within the SS gradient. HAEC were exposed to fluid SS for 24 hrs in a Hele-Shaw flow chamber, fixed, immunolabeled for microtubules, and counterstained with the nuclear stain DAPI. Cells were visualized using fluorescent microscopy and combined using a large image stitch. (**a**) Centerline microtubule intensity profile vs. SS with loess fit in a single monolayer. (**b**) DAPI positive cells were enumerated and plotted as a percent of the static condition. Mean ± SE, n=3-4.

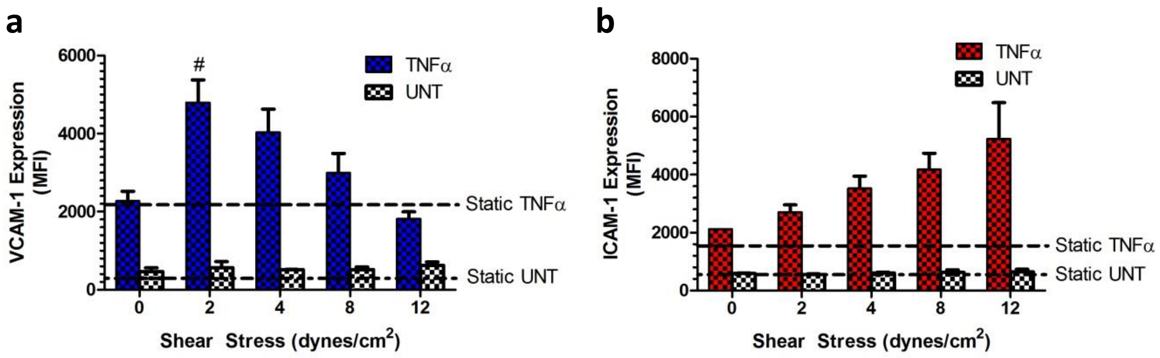

Supplement Figure 3: VCAM-1 and ICAM-1 are modulated by SS and TNFα. HAEC were exposed to fluid SS for 4 hrs in the presence or absence of TNFα. Control HAEC were treated with identical media in static culture dishes for 4 hrs. HAEC were then immunolabeled for (**a**) VCAM-1 and (**b**) ICAM-1 protein and fluorescently imaged. Mean ± SE, n=3-4. # P<0.05 vs static.

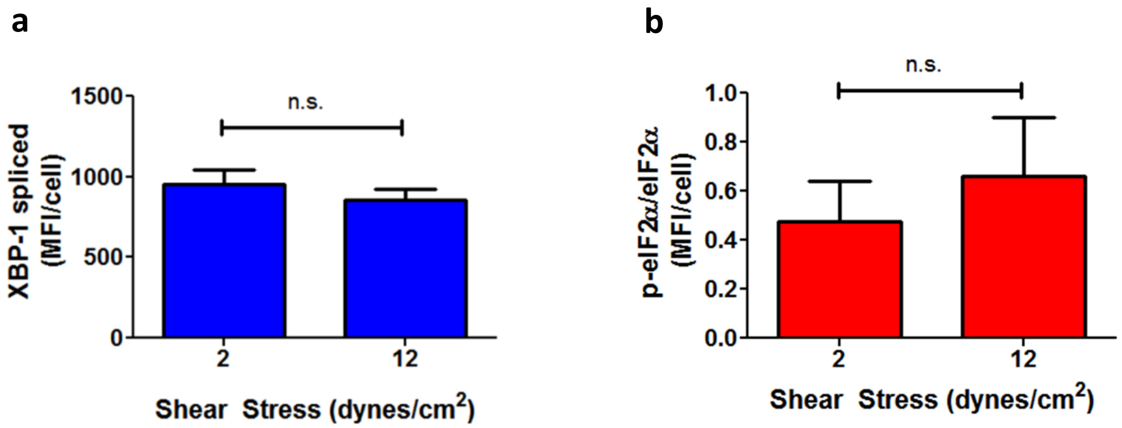

Supplement Figure 4: Exposure to SS alone does not elicit ER stress activation. HAEC were exposed to fluid SS for 24 hrs in a Hele-Shaw flow chamber and immunolabeled for the ER stress pathways proteins (**a**) XBP1 or (**b**) eIF2α and p-eIF2α. Mean ± SE, n=3-4.

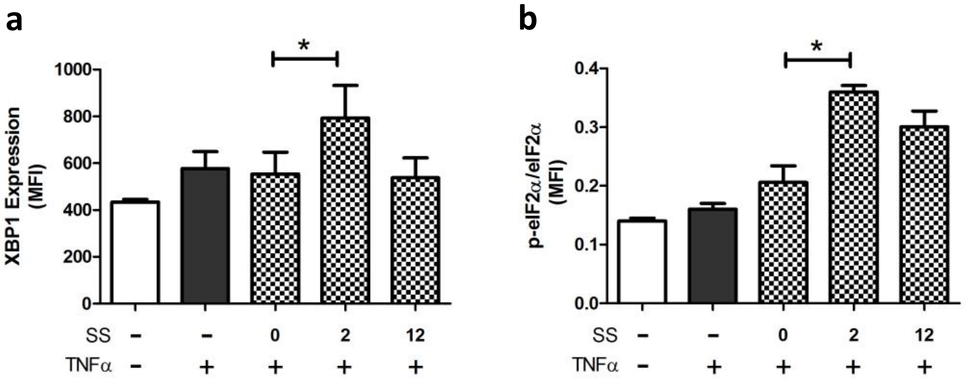

Supplement Figure 5: Dynamic range in ER stress activation. HAEC were treated in static culture dishes or exposed to fluid SS for 4 hrs in a Hele-Shaw flow chamber in the presence or absence of TNFα and immunolabeled for the ER stress pathways proteins (**a**) XBP1 or (**b**) eIF2α and p-eIF2α. Mean ± SE, n=3-4. * P<0.05.

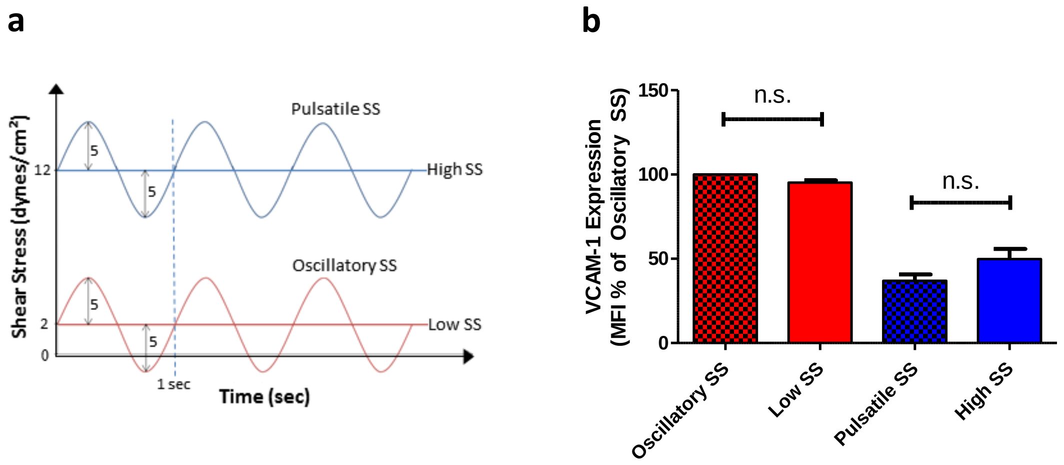

Supplement Figure 6: Acute VCAM-1 expression is dependent on the time-averaged magnitude of shear stress (SS). HAEC were simultaneously stimulated with TNFα (0.3 ng/ml) and exposed to fluid SS within parallel channels in a microfluidic chip for 4 hrs. (**a**) The SS profile was controlled using a programmable syringe pump (Oscillatory SS: 2 ± 5 dynes/cm² @ 1 Hz, Low SS: 2 dynes/cm², Pulsatile SS: 12 ± 5 dynes/cm² @ 1 Hz, High SS: 12 dynes/cm²). (**b**) VCAM-1 expression was quantified by immunofluorescence and normalized to Oscillatory SS. Mean ± SE, n=3, n.s. = not significant by Student's t-test.
